# Aquaporins: A new target for traditional Chinese medicine in the treatment of digestive system diseases

**DOI:** 10.3389/fphar.2022.1069310

**Published:** 2022-12-01

**Authors:** Yuchan Huang, Shidu Yan, Zixia Su, Lei Xia, Jinling Xie, Fan Zhang, Zhengcai Du, Xiaotao Hou, Jiagang Deng, Erwei Hao

**Affiliations:** ^1^ Guangxi Key Laboratory of Efficacy Study on Chinese Materia Medica, Guangxi University of Chinese Medicine, Nanning, China; ^2^ Guangxi Collaborative Innovation Center of Research on Functional Ingredients of Agricultural Residues, Guangxi University of Chinese Medicine, Nanning, China; ^3^ Guangxi Key Laboratory of TCM Formulas Theory and Transformation for Damp Diseases, Guangxi University of Chinese Medicine, Nanning, China

**Keywords:** aquaporin, gastrointestinal diseases, traditional Chinese medicine, water metabolism, triple energizer

## Abstract

Aquaporins (AQPs) are a family of transmembrane proteins expressed in various organ systems. Many studies have shown that the abnormal expression of AQPs is associated with gastrointestinal, skin, liver, kidneys, edema, cancer, and other diseases. The majority of AQPs are expressed in the digestive system and have important implications for the physiopathology of the gastrointestinal tract as well as other tissues and organs. AQP regulators can prevent and treat most gastrointestinal-related diseases, such as colorectal cancer, gastric ulcer, and gastric cancer. Although recent studies have proposed clinically relevant AQP-targeted therapies, such as the development of AQP inhibitors, clinical trials are still lacking and there are many difficulties. Traditional Chinese medicine (TCM) has been used in China for thousands of years to prevent, treat and diagnose diseases, and is under the guidance of Chinese medicine (CM) theory. Herein, we review the latest research on the regulation of AQPs by TCMs and their active components, including *Rhei Radix et Rhizoma*, Atractylodis macrocephalae Rhizoma, *Salviae miltiorrhizae Radix et Rhizoma*, *Poria*, *Astragali radix*, and another 26 TCMs, as well as active components, which include the active components include anthraquinones, saponins, polysaccharides, and flavonoid glycosides. Through our review and discussion of numerous studies, we attempt to explore the regulatory effects of TCMs and their active components on AQP expression in the corresponding parts of the body in terms of the Triple Energizer concept in Chinese medicine defined as “upper energizer, middle energizer, and lower energizer,”so as to offer unique opportunities for the development of AQP-related therapeutic drugs for digestive system diseases.

## 1 Introduction

Aquaporins (AQPs) are a family of water channels/proteins that play an important role in water transport and metabolism in the body. The first AQP was discovered on human erythrocyte membranes in the 1980s (Benga, 2003, 2006). In the 1990s, other AQPs were discovered in plants, microorganisms, various animals, and humans (Benga, 2009). AQPs are widely distributed in numerous organs (e.g., the gastrointestinal tract, liver, eye lens, brain, kidneys, and lungs) and tissues of the human body. AQPs are involved in water absorption and secretion in the body, maintaining an equilibrium in the amount of intra- and extracellular water; in addition, they are involved in other important physiological activities, such as cell proliferation, migration and angiogenesis (Nagaraju et al., 2016; Liu et al., 2021).

**FIGURE 1:**
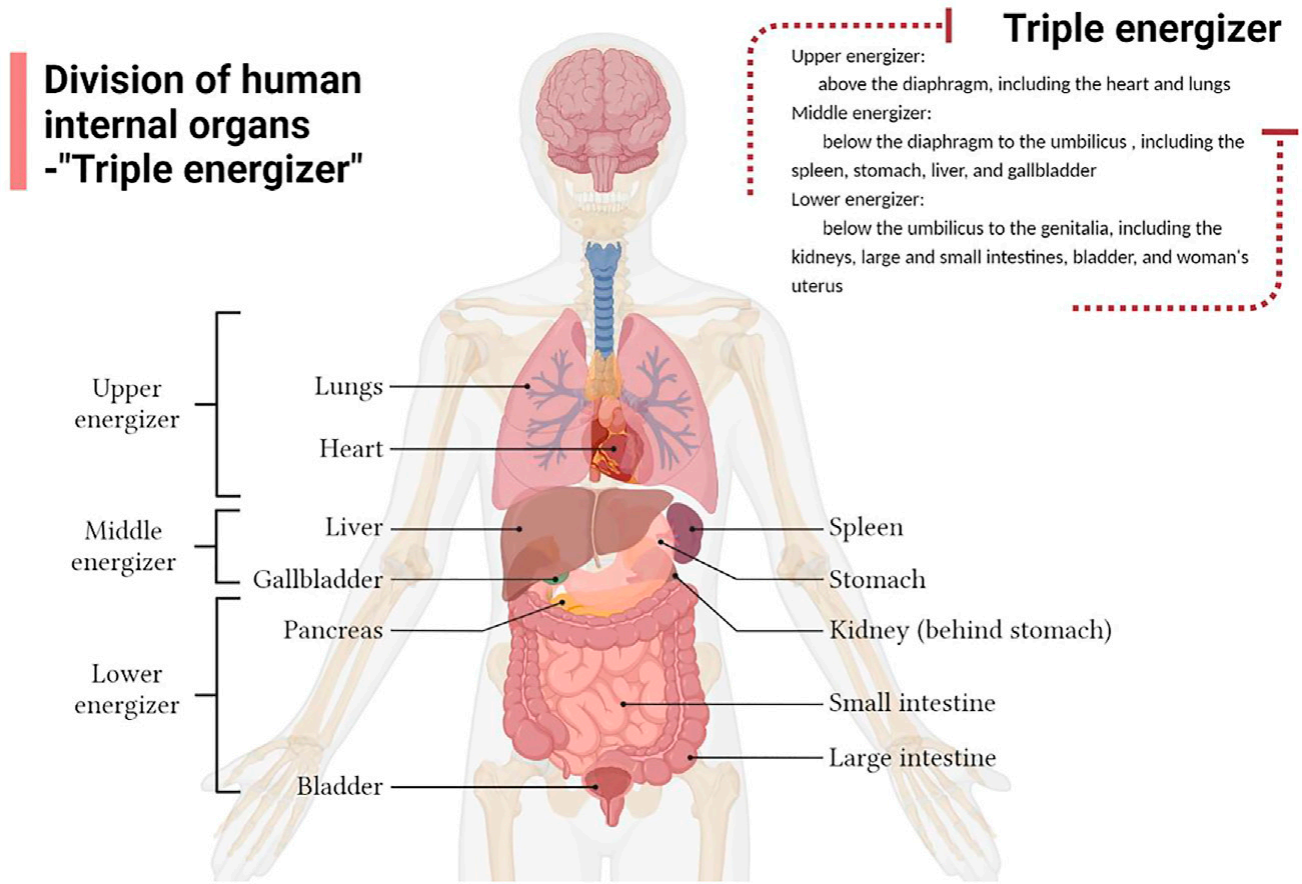
Division of the human internal organs according to the Triple Energizer concept of Traditional Chinese medicine.

The term “triple energizer” originates from the Yellow Emperor’s Inner Classic, which is the earliest traditional Chinese medical classic. And research proposes that the triple energizer organ is the largest tissue system linking all organs of the human body with a special tissue structure, and plays the role of a channel for stem cell reserve, water and fluid metabolism, immune regulation, and signal transmission (An et al., 2018). Many Chinese medical practitioners believe that above the diaphragm is the upper energizer, including the heart and lungs; below the diaphragm to the umbilicus is the middle energizer, including the spleen, stomach, liver, and gallbladder; and below the umbilicus up to the genitalia is the lower energizer, including the kidneys, large and small intestines, bladder, and woman’s uterus (Li et al., 2019a) (Figure 1). Water metabolism is the process of water production, transport, distribution and excretion. Chinese medicine (CM) theory suggests that water metabolism in the body involves a series of physiological functions of the viscera, particularly the coordination and cooperation of the “triple energizer” the lungs, spleen, kidneys, intestine, and bladder (Zhang et al., 2017b). Modern medicine believes that water metabolism requires AQPs, which are widely expressed in the spleen, lungs, kidneys, and digestive system and are closely related to water metabolism.

Currently, at least 11 AQPs have been found in the stomach, small and large intestines (Lv et al., 2022) (Table 1). AQP1 is abundantly distributed in the endothelial cells of the gastrointestinal tract. AQP3 and AQP4 are mainly distributed in the basolateral membrane of gastric and intestinal epithelial cells. AQP7, AQP8, AQP10, and AQP11 are distributed in the apical of enterocytes in the small and large intestines (Zhu et al., 2016). According to CM, digestive system diseases are caused by exogenous evil-qi, internal injuries, diet, emotions, and viscera dysfunction. The application of CM theory to TCMs and their compounds suggests that most TCMs and TCM compounds can treat digestive system diseases by reducing pathogenic bacteria, improving the intestinal mucosal barrier, and inhibiting intestinal and systemic inflammation, thus safeguarding people’s digestive health (Zhang et al., 2022). Therefore, studies investigating the regulatory mechanism of TCM and its components on AQPs will have a significant impact on the development of aquaporin-related therapeutic drugs.

**TABLE 1:** Location and pathologies in the gastrointestinal tract of AQPs.

AQPs	Stomach	Small intestine	Large intestine	Pathologies in the gastrointestinal tract	References
AQP0	*	*	*	Unknown	
AQP1	+	+	+	Gastric cancer	(Laforenza, (2012); Nagaraju et al. (2016); Shiozaki et al. (2021); Lv et al. (2022))
				Colon cancer	
				Pancreatic cancer	
AQP2	*	*	+	Unknown	Wang et al. (2018c)
AQP3	+	+	+	Diarrhea	(Laforenza, (2012); Nagaraju et al. (2016); Shiozaki et al. (2021); Yde et al. (2021); Lv et al. (2022))
				Constipation	
				Irritable bowel syndrome	
				Inflammatory bowel disease	
				Gastric cancer	
				Esophageal cancer	
AQP4	+	+	+	Colon inflammation	(Laforenza, (2012); Duan et al. (2013); Nagaraju et al. (2016); Zhang et al. (2017a); Wang et al. (2019); Lv et al. (2022))
				Diarrhea	
				Intestinal dysfunction	
				Gastric cancer	
AQP5	+	+	+	*Helicobacter pylori* infection	(Laforenza, (2012); Nagaraju et al. (2016); Zhu et al. (2017b); Li et al. (2021b); Lv et al. (2022))
				*Helicobacter* pylori-induced gastric carcinogenesis	
				Esophageal cancer	
				Colon cancer	
				Pancreatic cancer	
AQP6	*	+	*	Unknown	Lv et al. (2022)
AQP7	+	+	+	Diarrhea-predominant irritable bowel syndrome	(Laforenza, (2012); Camilleri et al. (2019); Lv et al. (2022))
AQP8	+	+	+	Colorectal cancer	(Laforenza, (2012); Zhu et al. (2017b); Magouliotis et al. (2020); Xie et al. (2021); Lv et al. (2022))
				Diarrhea-predominant irritable bowel syndrome	
				Inflammatory bowel disease	
AQP9	*	+	+	Inflammatory disease	(Ricanek et al. (2015); Lv et al. (2022))
AQP10	+	+	+	Diarrhea	(Laforenza, (2012); Wu et al. (2020); Lv et al. (2022))
AQP11	+	+	+	Unknown	(Laforenza, (2012); Lv et al. (2022))
AQP12	*	*	*	Unknown	

Note: “+” means there is expression, and “*” means no expression or unknown.

Research on the structure of the AQP family has been conducted, and many studies have reported on the various functions of AQPs in human health and disease (Ahmed and Ghafoor, 2021). The expression of AQPs is related to various diseases or pathological factors. It has been confirmed that AQP expression is involved in various pathological processes, such as intestinal, kidney, skin, and respiratory diseases and tumors. In the field of TCM, many scholars have conducted a series of studies on the correlation between AQPs and water metabolism. However, few studies have investigated the regulatory effects of TCMs and their main components on AQPs. Through their active components (e.g., anthraquinones, saponins, polysaccharides, and flavonoids), Rhei Radix et Rhizoma, Atractylodes macrocephala, Salviae miltiorrhizae Radix et Rhizoma, Poria, Scutellariae Radix, and Glycyrrhizae Radix et Rhizoma exert certain effects on diseases caused by water and electrolyte disorders by regulating the expression of AQPs. This review article summarizes the TCMs and their components that exert regulatory effects on AQPs. In addition, it provides a basis for further research on the treatment of AQP expression-related diseases through TCMs.

## 2 AQP overview

AQPs constitute a small and complete family of membrane proteins that promote water transport through the plasma membrane in response to osmotic gradients (Verkman et al., 2014). Each AQP has a structure comprising six transmembrane helices. According to the “hourglass model,” AQP monomers are arranged as tetramers in the plasma membrane: the four subunits are arranged in parallel, forming a fifth pore in the center of the tetramer (Kruse et al., 2006; Abir-Awan et al., 2019). Currently, 13 types of AQPs (AQP0–AQP12) have been described, which share the same characteristic amino acid sequence composed of the highly conserved motif asparagine-proline-alanine (Wittekindt and Dietl, 2019). AQPs are divided into three categories according to their functional characteristics. First, AQP0, AQP1, AQP2, AQP4, AQP5, and AQP8 are water-specific channels. Second, AQP3, AQP6, AQP7, AQP9, and AQP10 are permeable to water, urea, and glycerol. Finally, AQP11 and AQP12 are termed super AQPs because they share <15% structural homology with other AQPs (Cao et al., 2018; Xuan et al., 2021) (Table 2).

**TABLE 2:** AQPs: tissue distribution and involvement in pathologies.

AQP	Substrate	Main tissue distribution	Pathologies in which AQPs are implicated	References
AQP0	Water	Lens	Mutations in the gene for AQP0 often result in bilateral cataracts	(Chepelinsky, (2009); Wang et al. (2015); Abir-Awan et al. (2019); Wittekindt and Dietl, (2019))
AQP1	Water Glycerin	Erythrocytes	Deficiency leads to inability to concentrate in urine, tumor metastasis, and increased aggressiveness to alter pain	(Murata et al. (2000); Oshio et al. (2005); Wang et al. (2015); Zhu et al. (2016); Nakano et al. (2018); Abir-Awan et al. (2019); Wittekindt and Dietl, (2019))
		Choroid plexus		
		Eye epithelium		
		Corneal epithelium		
		Bile duct		
		Capillaries Nephron		
		Airways		
		Skeletal muscle		
		Endothelial cells of the gastrointestinal tract		
AQP2	Water	Collecting duct cells	Deficiency causes diabetes insipidus	(Kwon et al. (2013); Wang et al. (2015); Vukićević et al. (2016); Abir-Awan et al. (2019); Wittekindt and Dietl, (2019))
AQP3	Water	Epidermis Collecting duct cells	Skin dehydration	(Boury-Jamot et al. (2006); Hara-Chikuma et al. (2015); Wang et al. (2015); Abir-Awan et al. (2019); Wittekindt and Dietl, (2019); Deng et al. (2021); Tricarico et al. (2022))
	Glycerol	Erythrocytes	Psoriasis	
	Urea	Gastric and intestinal epithelial cells	Tumor invasiveness and metastases	
AQP4	Water	Astrocytes	Cytotoxic edema	(Nagelhus and Ottersen, (2013); Wang et al. (2015); Zhu et al. (2016); Abir-Awan et al. (2019); Wittekindt and Dietl, (2019); Deng et al. (2021))
		Parietal cells	Vasogenic edema Neuromyelitis optica	
		Collecting duct cells	Glioblastoma	
		Retina	Alzheimer’s disease	
		Gastric parietal cells		
		Basolateral membrane of gastric and intestinal epithelial cells		
AQP5	Water	Salivary, lacrimal, and sweat glands	Sjögren’s syndrome	(Verkman et al. (2000); Day et al. (2014); Wang et al. (2015); Abir-Awan et al. (2019); Wittekindt and Dietl, (2019); Huang et al. (2020a))
		Lung	Tumors	
		Kidney		
		Colon		
AQP6	Water	Collecting duct	Tumors	(Wang et al. (2015); Jang et al. (2016); Ma et al. (2016); Wittekindt and Dietl, (2019))
	Glycerol	Retina		
	Urea			
AQP7	Water	Adipocytes	Adipocyte hypertrophy	(Wang et al. (2015); Zhu et al. (2016); Iena and Lebeck, (2018); Abir-Awan et al. (2019); Wittekindt and Dietl, (2019); Huang et al. (2022))
	Glycerol	Testis		
	Urea	Proximal kidney tubule		
		Apical of enterocytes in the small and large intestines		
AQP8	Water	Pancreas	Tumors	(Li et al. (2008); Wang et al. (2015); Zhu et al. (2016); Abir-Awan et al. (2019); Wittekindt and Dietl, (2019); Deng et al. (2021))
	Urea	Testis	Colorectal cancer	
		Apical of enterocytes in the small and large intestines		
AQP9	Water	Hepatocytes	Osteoporosis	(Jelen et al. (2011); Calamita et al. (2012); Wang et al. (2015); Abir-Awan et al. (2019); Wittekindt and Dietl, (2019))
	Glycerol	Osteoclasts		
	Urea	Astrocytes		
		Leukocytes		
AQP10	Water	Intestinal epithelial cells	Obesity	(Laforenza et al. (2013); Wang et al. (2015); Abir-Awan et al. (2019); Wittekindt and Dietl, (2019); Huang et al. (2022))
	Glycerol	Adipocytes		
	Urea			
AQP11	Water	Testis	Polycystic kidney disease	(Wang et al. (2015); Koike et al. (2016); Tanaka et al. (2016); Zhu et al. (2016); Abir-Awan et al. (2019); Wittekindt and Dietl, (2019))
	Glycerol	Thymus		
		Kidney		
		Liver		
		Apical of enterocytes in the small and large intestines		
AQP12	Uncertain	Pancreatic acinar cells	Unknown	(Wang et al. (2015); Abir-Awan et al. (2019); Wittekindt and Dietl, (2019); da Silva et al. (2020))

## 3 Classification of AQPs and their involvement in different pathologies

### 3.1 AQP1

AQP1 was first discovered in erythrocytes and renal proximal tubules; it has been associated with pathological factors, such as cancer, inability to concentrate urine, and altered pain perception (Murata et al., 2000; Zou et al., 2013; Nave et al., 2016; Nakano et al., 2018; Cao et al., 2018). The expression of AQP1 in certain tumor types is associated with increased angiogenesis, invasion, metastasis, and proliferation (Verkman et al., 2014; Nave et al., 2016). Studies revealed that AQP1 is expressed in peripheral vascular endothelial cells and is involved in tumor angiogenesis. It is upregulated by angiogenic factors during hypoxia and is required for endothelial cell migration and angiogenesis (Nave et al., 2016; De Ieso and Yool, 2018). There is also evidence that AQP1 is expressed in choroid plexus epithelial cells and is closely related to the formation of cerebrospinal fluid (Oshio et al., 2005). Therefore, effective regulation of AQP1 plays an important role in the treatment of cancers.

### 3.2 AQP2

AQP2 plays an important role in regulating the balance of water and other solutes in the body and is also a key protein that controls the permeability of the renal collecting duct (Kwon et al., 2013; Vukićević et al., 2016). Following an increase in plasma osmolality, the neurohypophysial hormone arginine vasopressin is secreted and binds to arginine vasopressin receptor 2 (AVPR2) in the principal cells of the renal collecting duct. This leads to the insertion of AQP2 in the apical plasma membrane, which is responsible for water reabsorption (Hj and Th, 2016; Ouyang et al., 2020). Dysregulation of AQP2 is closely associated with several clinical disorders characterized by disturbances in body water balance, including congenital nephrogenic diabetes insipidus (Li Q. et al., 2021), lithium-induced nephrogenic diabetes insipidus, electrolyte disturbances, acute and chronic renal function failure, ureteral obstruction, nephrotic syndrome, congestive heart failure, and liver cirrhosis (Kwon et al., 2013).

### 3.3 AQP3

AQP3 is the most abundant aqua-glycerol-porin in the skin. Boury-Jamot et al. confirmed that AQP3 is expressed on the plasma membrane of human keratinocytes (Boury-Jamot et al., 2006). Studies have found that the overexpression of AQP3 is associated with skin malignancies, such as melanoma (Hara-Chikuma and Verkman, 2008). Hara-Chikuma et al. demonstrated that H_2_O_2_ is imported through AQP3 and participates in nuclear factor-κB (NF-κB) signal transduction in keratinocytes (Hara-Chikuma et al., 2015). Furthermore, AQP3 is involved in the pathogenesis of psoriasis. Recent experimental findings on whether systemic AQP3 deficiency causes vitiligo suggest that both systemic and localized AQP3 deficiency exists in vitiligo and correlates with disease severity and oxidative stress (Chandrashekar et al., 2021). In general, numerous studies have shown that AQP3 is closely related to human skin diseases and is of great significance for treatment.

### 3.4 AQP4

Compared with other AQPs, AQP4 is characterized by relatively high single-channel water permeability (Yang et al., 1997). Similar to AQP1, AQP4 is the major water channel in the central nervous system and is considered a major regulator of cerebral fluid homeostasis in both healthy and pathophysiological conditions (Trillo-Contreras et al., 2019). It is mainly expressed in astrocytes (Nagelhus and Ottersen, 2013). AQP4 plays an important role in the pathology of brain diseases. Analysis of AQP4 knockout mice showed that the AQP4 gene is widely involved in brain water balance, neural excitation, glial scarring, neuroinflammation, neurodegeneration, and neurological mental illness (Verkman et al., 2017). Lan et al. confirmed that AQP4 may affect astrocyte calcium signaling and potassium homeostasis in the pathogenesis of Alzheimer’s disease (Lan et al., 2017). Moreover, AQP4 is related to the removal of interstitial β-amyloid and glutamate neurotransmission. Thus, an in-depth study of AQP4 is necessary to provide a reference for the treatment of various diseases.

### 3.5 AQP5

AQP5 is present in airway epithelial cells, type I alveolar epithelial cells, and lung submucosal acinar cells (Verkman et al., 2000). It plays a role in many secretory glands (e.g., salivary, lacrimal, and sweat) (Day et al., 2014; Wang et al., 2020a), and is key in water transport. The high expression of AQP5 in breast cancer cells affects their invasive ability, proliferation, and metastatic potential (Jung et al., 2011). Downregulation of AQP5 expression can inhibit the proliferation and migration of ectopic endometrial epithelial cells, as well as the formation of endometrial cell nodules in nude mice (Xin et al., 2015). This evidence also suggests that the alteration of AQP5 expression is linked to tumor progression.

### 3.6 AQP0, 6–12

Originally, AQP0 was considered to be a membrane endogenous protein specifically expressed in lens fibers. It is the main endogenous protein of the lens termed MIP. Mutations and knockouts of AQP0 can lead to the development of lens cataracts. AQP0 functions as a water channel and adhesion molecule in lens fibers, resulting in a narrowing of the intercellular space of lens fibers, which is required for lens transparency and accommodation (Chepelinsky, 2009). AQP6 is selectively localized to the ganglion cell layer and outer plexiform layer in the rat retina (Jang et al., 2016); additionally, it is highly expressed in benign ovarian tumors (Ma et al., 2016). AQP7 is the most representative glycerol channel protein expressed in pancreatic β cells. It is involved in insulin secretion, triacylglycerol synthesis, and the proliferation of endocrine cells (Méndez-Giménez et al., 2018; Arsenijevic et al., 2019). Iena and Lebeck, 2018 found that AQP7 deficiency was associated with increased glycerol kinase activity and triglyceride accumulation in adipose tissue, leading to the secondary development of obesity and insulin resistance.

AQP8 is expressed in a variety of cancers (e.g., colorectal, breast, lung, and prostate) (Wu et al., 2018). AQP9 is a major factor in the pathway for glycerol uptake by hepatocytes (Jelen et al., 2011; Calamita et al., 2012). AQP9 is found in astrocytic processes and cell bodies and pathophysiologic conditions may regulate AQP9 expression (Badaut et al., 2002). Laforenza et al. showed that AQP10 is another glycerol channel expressed on the plasma membrane of human adipocytes (Laforenza et al., 2013). AQP11 is expressed in the testes, thymus, kidneys, liver, and intestines (Koike et al., 2016). Studies have shown that mice lacking AQP11 can develop polycystic kidney disease (Tanaka et al., 2016). The expression of AQP12 is associated with lower levels of pro-inflammatory cytokines, higher levels of anti-inflammatory cytokines, and the promotion of insulin secretion (da Silva et al., 2020).

## 4. AQP regulation by TCMs and their components

### 4.1 Purgative medicinals

Medicinals with the main efficacy of promoting defecation by purgation are known as purgative medicinals (Zhang and Ye, 2020).

#### 4.1.1 Rhei Radix et Rhizoma (*Rheum palmatum* L. or *Rheum tanguticum* Maxim. ex Balf. or *Rheum officinale* Baill.)

Rhei Radix et Rhizoma (State Pharmacopoeia Commission., 2020; Zhang and Ye, 2020) is the main purgative medicine, which acts on the spleen, stomach, large intestine, and liver meridians and exerts various pharmacological effects (e.g., purgative, antipyretic, antibacterial, diuretic, and choleretic). It also can act on upper energizer, middle energizer, and lower energizer. The major purgative site of Rhei Radix et Rhizoma is the colon (LI et al., 2008). Bao et al. (2021) established a model of diarrhea in mice by gavage of Rhei Radix et Rhizoma. The results showed that Rhei Radix et Rhizoma can lead to the downregulation of AQP3 and AQP4 expression, reduce the absorption of water in the colon, and increase the water content of the intestinal tract. Wang et al. (2021) analyzed the metabolites of rhubarb anthraquinone and its chemical marker sennoside A in a Rhei Radix et Rhizoma diarrhea model. The results showed that rhubarb anthraquinone could downregulate the expression of various AQPs in the colon, kidneys, and liver of rats with diarrhea. Sennoside A, a component in Rhei Radix et Rhizoma, can reduce the expression of AQP3 in the colon, thereby inhibiting the transport of water from the intestinal lumen to the vascular side and exerting a laxative effect (Kon et al., 2014). Peng et al. (2017) showed that emodin can reduce the release of inflammatory factors, downregulate the expression of AQP4, reduce edema around the brain tissue after intracerebral hemorrhage, reduce the apoptosis of neurons, and promote the repair of nerve function. Kang et al. (2017) have found that Dahuang-Fuzi-Tang could ameliorate pulmonary and intestinal edema and injury induced by severe acute pancreatitis by up-regulating different AQPs in the lungs and intestines.

#### 4.1.2 Sennae Folium (*Cassia angustifolia* Vahl or *Cassia angustifolia* Delile)

Cao et al. prepared senna leaf extract (SE), sennoside, and sennoside A, and used them to establish a rat model of diarrhea (Cao et al., 2018). Using quantitative reverse-transcription polymerase chain reaction (RT-PCR), they determined and analyzed the expression profiles of AQP in six organs, namely the colon, kidneys, liver, spleen, lungs, and stomach. It was found that the expression of AQPs in the kidneys and liver of rats in the SE group was upregulated, whereas that of AQPs in the colon of the rats in the sennoside group was significantly downregulated. These findings indicated that SE and sennoside exert important effects on the regulation of AQPs.

#### 4.1.3 Phytolaccae Radix (*Phytolacca acinosa* Roxb. or *Phytolacca americana* L.)

Esculentoside A is the most abundant triterpenoid saponin in Phytolaccae Radix (Cui et al., 2014), and has immunomodulatory, anti-inflammatory, anti-glomerular sclerosis, and diuretic pharmacological effects. Li et al. (2015) investigated the regulatory effect of esculentoside A on the expression of AQP2 and AQP4 in the kidneys of rats treated with water loading. The results showed that esculentoside A reduced the reabsorption of water by downregulating the expression of AQP2, AQP4, and their mRNA levels in the kidneys of rats to achieve a diuretic effect.

### 4.2 Supplementation medicinals

Medicinals with the major efficacies of supplementing the deficiency, reinforcing weakness, supplementing qi, blood, yin, and yang are known as supplementation medicinals (Zhang and Ye, 2020).

#### 4.2.1 Atractylodis macrocephalae Rhizoma (*Atractylodes macrocephala* Koidz.)

Studies using slow transit constipation (STC) rats as an animal model found that the expression of AQP3 in the colon of STC rats was reduced and AQP4 was increased compared with that of normal rats. These results suggested that the treatment of Atractylodis macrocephalae Rhizoma on STC may be associated with the inhibition of AQP4 expression and the increase of AQP3 expression (Chen et al., 2019). Atractylenolide I, the main component of Atractylodis macrocephalae Rhizoma, can regulate the expression of AQP3 and AQP4 in the colon of STC rats and reduce intestinal water absorption (Jiang, 2017). Zhang concluded that the mechanism underlying the effects of Atractylodis macrocephalae Rhizoma in the treatment of spleen deficiency and diarrhea may be the regulation of AQP3 expression (Zhang, 2012). Through this effect, the Atractylodis macrocephalae Rhizoma improves the function of intestinal water metabolism. Xia et al. (2022) found that the volatile oil of Atractylodis macrocephalae Rhizoma and Ginseng Radix et Rhizoma could up-regulate the expression of AQP3 and AQP4 to treat chronic atrophic gastritis in rats.

#### 4.2.2 Astragali radix (*Astragalus membranaceus* (Fisch.) Bge. Var. *mongholicus* (Bge.) Hsiao or *Astragalus membranaceus* (Fisch.) Bge.)

Astragali radix mainly contains astragalus polysaccharides, saponins, and flavonoids, as well as other active components (Lv et al., 2021). Astragali radix mainly acts on upper energizer and middle energizer. Astragali radix is a classic drug mainly used for the treatment of digestive tract-related inflammation and tumors (Sun R.-L. et al., 2021). Mao et al. (2010) prepared a diabetic rat model by intraperitoneal injection of streptozotocin. The results demonstrated that astragalus polysaccharide can downregulate the overexpression of AQP2 in the renal medulla, relieve water and sodium retention in diabetes mellitus, and protect the kidneys. Zhao, 2009 used immunohistochemistry and western blotting to examine the changes in AQP1 and AQP5 expression in the lungs of acute lung injury (ALI) rats induced by bacterial endotoxin. Alphaside upregulated the expression of AQP1 and AQP5 in the lungs of ALI rats. Li et al. (2016) established an oxygen-glucose deprivation and reoxygenation model and pointed out that astragaloside I may affect the expression of AQP4 by downregulating the expression of tumor necrosis factor-α (TNF-α), thereby affecting astrocyte edema.

#### 4.2.3 Ginseng Radix et Rhizoma (*Panax ginseng* C.A. Mey.)

Ginseng Radix et Rhizoma contains the main components ginsenosides, ginseng polysaccharides, ginseng volatile oil and other trace elements, proteins, amino acids, vitamins, and other types of compounds. Modern medical research has shown that Ginseng Radix et Rhizoma can improve human immunity and cardiovascular disease, and exerts anti-aging, anti-tumor, hypoglycemic, and other effects (Wang, 2019). Studies found that ginsenoside Rg3 can reduce the levels of inflammatory mediators in the peripheral blood of rectal polyp model rats, as well as increase the expression of AQP3 and AQP4 in rectal polyp tissue (Xie et al., 2019a; 2019b). The use of panaxadiol saponins in severe acute pancreatitis can inhibit the expression of TNF-α and interleukin-6 (IL-6), reduce blood concentration, improve microcirculation, and upregulate the expression of AQP1 in lung tissue, thereby reducing pulmonary edema and lung injury (Zhao et al., 2009). Ginsenoside Rb1 can reduce body weight and improve glucose and lipid metabolism by upregulating the peroxisome proliferator-activated receptor gamma (PPARγ) and AQP7 protein levels (Guo et al., 2020). It can also relieve spinal cord edema and improve neurological function by increasing the expression of AQP4 (Li Y.-N. et al., 2019).

#### 4.2.4 Glycyrrhizae Radix et Rhizoma (*Glycyrrhiza uralensis* Fisch. or *Glycyrrhiza inflata* Bat. or *Glycyrrhiza glabra* L.)

The main components of Glycyrrhizae Radix et Rhizoma include flavonoids, triterpenes, and alkaloids; these components include the pentacyclic triterpenoid 18β-glycyrrhetinic acid. Liu et al., 2020 established a model of allergic rhinitis (AR) in rats by sensitization with ovalbumin. A therapeutic effect against AR in rats may be achieved by changing the content of inflammatory factors, increasing the expression of AQP5 in the nasal mucosa, and reducing the expression of mucin 5AC, oligomeric mucus/gel-forming.

#### 4.2.5 Angelicae Sinensis Radix (*Angellica sinensis* (Oliv.) Diels)

Angelicae Sinensis Radix, a traditional medicinal plant, is mainly used for nourishing blood, relieving pain, moistening the intestines, and treating irregular menstruation and amenorrhea in women (Wei et al., 2016). N-butylidenephthalide is a bioactive compound in Angelicae Sinensis Radix. Liao et al. have found that N-butylidenephthalide caused gastric cancer cell death through the activation of the mitochondria-intrinsic pathway and induced the REDD1 expression leading to mTOR signal pathway inhibition in gastric cancer cells (Liao et al., 2018). Du et al. explored the mechanism of action of Angelicae Sinensis Radix and laxative through a mouse model of blood deficiency and constipation (Du et al., 2018a, 2017; 2018b). They concluded that three doses of Angelicae Sinensis Radix decoction can significantly downregulate the expression of AQP4 and AQP8 in the colon. The mechanism underlying this effect may be related to the regulation of the colon phospholipase-C-inositol 1,4,5-trisphosphate-calmodulin (PLC-IP3-CaM) signaling pathway and the adenylate cyclase/cyclic adenosine monophosphate/protein kinase A (AC-cAMP-PKA) signaling pathway by Angelicae Sinensis Radix, thereby reducing the reabsorption of colonic water. Angelicae polysaccharide and angelica volatile oil may be important components of Angelicae Sinensis Radix for exerting its “laxative” effect (Du et al., 2018c). Researchers established a mouse model of asthma and concluded that Angelicae Sinensis Radix can promote the expression of AQP1 and its gene in lung tissue, thereby exerting a certain therapeutic effect on Yin deficiency asthma (Wang et al., 2016).

#### 4.2.6 Paeoniae Radix Alba (*Paeonia lactiflora* Pall.)

Modern pharmacological studies have found that total glucosides of Paeoniae Radix Alba, the active component of Paeoniae Radix Alba, can enhance the amplitude of colon contraction and prolong the contraction time (Yang et al., 2002). Fang and Gao, 2017 explored the laxative effect and mechanism of Paeoniae Radix Alba by establishing an STC mouse model. The results showed that high and medium doses of Paeoniae Radix Alba could significantly reduce the expression of AQP4 in the colon of STC mice and enhance the intestinal water to achieve a laxative effect.

#### 4.2.7 Cistanches Herba (*Cistanche deserticola* Y. C. Ma or *Cistanche tubulosa* (Schenk) Wight)

Cistanches Herba mainly acts on lower energizer. Tao et al., 2015 suggested that phenylethanoid glycosides from Cistanche can reduce the water content of brain tissue and the expression of AQP4 mRNA in the brain tissue of rats presenting high-altitude cerebral edema. These findings indicated that phenylethanoid glycosides from Cistanche can prevent the occurrence of high-altitude cerebral edema, and this effect may be related to the inhibition of AQP4 expression. Cistanches Herba can simultaneously regulate AQP1 in the lungs and kidneys and enhance the regulatory effect on water metabolism (Zhang et al., 2012).

#### 4.2.8 Ophiopogonis Radix (Ophiopogon japonicus (L.f) Ker-Gawl.)

Researchers have established a model of Sjögren’s syndrome (SS) mice to investigate the mechanism through which Ophiopogon japonicus polysaccharide can regulate the inflammatory microenvironment of the submandibular gland in SS. Using this model, Ophiopogon japonicus polysaccharide could significantly increase the expression levels of AQP5, thereby regulating the submandibular gland inflammatory microenvironment and improving SS (Tao et al., 2014). Sun et al. showed that the effective components of Ophiopogonis Radix (polysaccharides and saponins) could upregulate the expression of AQP1 and AQP5 in the lung tissue of mice with Mycoplasma pneumonia, reduce the pathological changes in lung tissue, and play a role in moisturizing the lungs and producing fluid (Sun Y. et al., 2021).

### 4.3 Damp-draining diuretic medicinals

Medicinals with the main functions of regulating water passages, as well as draining water and dampness are called damp-draining diuretic medicinals (Zhang and Ye, 2020).

#### 4.3.1 Poria (*Poria cocos* (Schw.) Wolf)

The main active components of Poria (i.e., polysaccharides and triterpenes) are commonly used clinically to treat dysuria, edema, diarrhea due to spleen deficiency, loose stools, palpitations, and insomnia (Li et al., 2022). Li et al. 2021a analyzed the dampness effect of Poria from different origins on kidney-Yang deficiency and lower energizer edema in rats. The levels of AQP1 and AQP2 in the renal medulla of the rats in each administration group were decreased; the serum anti-diuretic hormone levels were also significantly decreased. These results indicated that Poria may regulate AQP1- and anti-diuretic hormone-AQP2-related pathways to achieve its diuretic and osmotic effects. Zhang established a model of diarrhea due to spleen deficiency in rats and studied the regulatory effect of Poria on water metabolism by measuring the expression levels of AQP3 and AQP4 in the jejunum and colon mucosa (Zhang, 2012). The results indicated that Poria may enhance the reabsorption of water in colonic mucosa by upregulating the expression of AQP3 in colonic mucosa.

#### 4.3.2 Alismatis Rhizoma (*Alisma orientale* (Sam.) Juzep. or *Alisma plantago-aquatica* Linn.)

Alismatis Rhizoma facilitates urination and clears dampness and heat, and mainly acts on lower energizer. Wu et al. (2010) observed the relative expression of AQP2 mRNA in the renal medulla using RT-PCR after administration of the water extract of Alismatis Rhizoma in rats. The results showed that the AQP2 mRNA expression could be significantly reduced after eight consecutive administrations of Alismatis Rhizoma water extract to rats, indicating that the diuretic activity of Alismatis Rhizoma may be produced by regulating the reduction of AQP2 expression in the renal medulla. Ding et al. have found that Qutanhuoxue Decoction could upregulate AQP7 expression in adipocytes and downregulate AQP9 expression in hepatocytes to treat Nonalcoholic fatty liver disease (Zheng et al., 2019). And the composition of Qutanhuoxue Decoction inclouding Poria, Alismatis Rhizoma, Astragali radix, Coicis Semen, Bupleuri Radix, and another 6 TCMs.

#### 4.3.3 Polyporus (*Polyporus umbellatus* (Pers.) Fries)

Zhang et al. confirmed that the water extract of Polyporus sclerotiorum and Polyporus polysaccharide exerts obvious inhibitory effects on bladder cancer (Zhang et al., 2011). Researchers used Polyporus, combining N-butyl-N-(4-hydroxybutyl)-nitrosamine with saccharin, to establish a bladder cancer model in Fisher-344 rats (Zhang and Zhang, 2013). The expression of AQP1 and AQP3 in bladder cancer tissue was measured by immunohistochemistry and RT-PCR. The results showed that Polyporus could significantly downregulate the relative expression of AQP1 and AQP3 mRNA in bladder cancer tissue of rats, indicating that the anti-bladder cancer effect may be produced by regulating AQP1 and AQP3 in bladder cancer tissue.

#### 4.3.4 Coicis Semen (*Coix lactyma-jobi* L.var.ma-yuen (Roman.) Stapf)

Coixenopolysaccharide is the most abundant functional component in Coicis Semen. Wang Y. et al., 2018 explored the mechanism of Coixenopolysaccharide underlying the regulation of water metabolism in rats with dampness stagnancy due to spleen deficiency by replicating the rat model of spleen deficiency. The results showed that Coixenopolysaccharide upregulated the expression of AQP3 in colon tissue. The regulatory effect of Coixenopolysaccharide on AQP3 may be one of the mechanisms involved in the treatment of spleen deficiency and dampness in rats.

#### 4.3.5 Plantaginis Semen (Plantago asiatica L. Or *Plantago depressa* Willd.)

Yan et al. investigated the diuretic activity of Plantaginis Semen and reduced the expression of AQP2 and AQP1 in the renal medulla through a model of rats treated with water loading (Yan et al., 2014). Wang et al. used Sennae Folium to replicate the model of diarrhea in rats and studied the effect of Plantaginis Semen on the expression of AQP1 mRNA and protein in the small intestine of rats with diarrhea (Wang et al., 2020c). The results showed that the mechanism of Plantaginis Semen involved in relieving diarrhea is related to the upregulation of AQP1 expression, an increase in the absorption of water in the small intestine, and the promotion of water metabolism. Wang et al. showed that the mechanism may be related to the inhibition of the inflammatory response, repair of pathological damage to the colonic mucosa, and upregulation of AQP4 expression (Wang et al., 2020b).

### 4.4 Blood-stanching medicinals

Medicinals that can stop various forms of bleeding, internal or external, are called blood-stanching medicinals (Zhang and Ye, 2020).

#### 4.4.1 Notoginseng Radix et Rhizoma (*Panax notoginseng* (Burk.) F. H. Chen).

Panax notoginseng saponins (PNS) are the main pharmacologically active components of Notoginseng Radix et Rhizoma. Li et al. found that PNS could inhibit the expression of caspase 1 (CASP1) and CASP3 proteins after cerebral ischemia-reperfusion, thereby playing a role in the protection of the brain (Li et al., 2006). Meng et al. measured the expression of AQP4 protein and mRNA in rats with cerebral hemorrhage by immunohistochemical staining and RT-PCR, respectively (Meng et al., 2007). It is hypothesized that PNS may reduce the formation of cerebral edema after intracerebral hemorrhage by inhibiting the expression of AQP4.

### 4.5 Blood-invigorating and stasis-dissolving medicinals

Medicinals with the main efficacy of facilitating blood vessels, promoting blood circulation and removing blood stasis are called blood-invigorating and stasis-dissolving medicinals (Zhang and Ye, 2020).

#### 4.5.1 Salviae miltiorrhizae Radix et Rhizoma (*Salvia miltiorrhiza* Bge.)

Salviae miltiorrhizae Radix et Rhizoma is commonly used in TCM to promote blood circulation and remove blood stasis. It eliminates blood stasis and improves microcirculation by dilating blood vessels and accelerating blood flow (Wang et al., 2015). Hu et al. reported that it can significantly reduce the expression of AQP2 in the kidney tissue of diabetic rats and increase the expression of AQP1 in the lungs (Hu et al., 2011). It is hypothesized that the protective effect of Salviae miltiorrhizae Radix et Rhizoma on the kidneys of diabetic rats may be related to the regulation of AQP1 and AQP2. Other studies demonstrated that tanshinone alleviated cerebral edema caused by brain injury through the inhibition of AQP4 induction by inflammatory factors (Huang Y. et al., 2020; Tang et al., 2022). In ALI and chronic lung injury, Salviae Miltiorrhizae Radix et Rhizoma can increase the expression of AQP1 in pulmonary capillaries, improve blood rheology, alleviate pathological changes in lung tissue, and alleviate the occurrence and development of lung injury to a certain extent (Li et al., 2009).

#### 4.5.2 Curcumae Longae Rhizoma (*Curcuma Longa* L.)

The main active components of Curcumae Longae Rhizoma are curcumin and volatile oil, which have anti-inflammatory, hypolipidemic, and anti-tumor properties (Ghandadi and Sahebkar, 2017). Lin established a model of focal cerebral ischemia-reperfusion in rats. Treatment with curcumin downregulated the expression of vascular endothelial growth factor (VEGF) and AQP4 (Lin, 2015). These findings indicated that the protective effect of curcumin on the cerebral ischemia-reperfusion injury was related to the downregulation of VEGF and AQP4 expression and alteration of the permeability of the blood-brain barrier (BBB).

### 4.6 Exterior-releasing medicinals

Medicinals that can disperse exterior pathogens are known as exterior-releasing medicinals (Zhang and Ye, 2020).

#### 4.6.1 Puerariae Lobatae Radix (*Pueraria Lobata* (Willd.) Ohwi)

Puerarin is a flavonoid glycoside isolated from the root of the TCM Puerariae Lobatae Radix. Many researchers found that puerarin has a protective effect on ischemia-reperfusion injury of the heart, brain, spinal cord, lung, intestine, and other organs (Gao et al., 2022). Studies have shown that puerarin may reduce the water channel function of AQP4 by regulating the cAMP/PKA/AQP4 pathway, thereby reducing subarachnoid hydrops (Ying and Liu, 2020). It may also inhibit the release of TNF-α and the phosphorylation of NF-κB and mitogen-activated protein kinase (MAPK) pathways, thereby inhibiting the upregulation of AQP4 expression during hypobaric hypoxia (Wang C. et al., 2018). In addition, puerarin affects the water transport of esophageal cancer cells by upregulating the expression of AQP1 mRNA (Jiang et al., 2011).

#### 4.6.2 Bupleuri Radix (*Bupleurum chinense* DC. or *Bupleurum scorzonerifolium* Willd.)

Saikosaponin a (SSa) is the main active component of Bupleurum Radix, exerting extensive pharmacological effects (e.g., anti-inflammatory, immunomodulatory, and antibacterial). Zhu et al. used the modified Allen heavy blow method to create a model of acute spinal cord injury in rats and explored the possible impact mechanism of SSa on the early stage of the injury (Zhu et al., 2017a). The results indicated that SSa may inhibit the NF-κB signaling pathway and the expression of AQP4. Moreover, it can reduce the inflammatory response and tissue edema after acute spinal cord injury, thus protecting the nerves.

#### 4.6.3 Asari Radix et Rhizoma (*Asarum heterotropoides* Fr. Schmidt var. *mandshuricum* (Maxim.) Kitag. or *Asarum sieboldii* Miq. var. *seoulense* Nakai or *Asarum sieboldii* Miq.)

The main component of Asari Radix et Rhizoma volatile oil is methyl eugenol. Wu et al. (2019) . established a model of AR in rats to observe the effect of methyl eugenol on the expression of AQP in the nasal mucosa. The results showed that methyl eugenol could upregulate the expression of AQP5 in the nasal mucosa of rats, thereby regulating the secretions by nasal mucosa glands in rats with AR and relieving their nasal symptoms.

### 4.7 Heat-clearing medicinals

Medicinals with the main function of clearing interior heat are called heat-clearing medicinals, which are usually to treat interior heat syndrome (Zhang and Ye, 2020).

#### 4.7.1 Cassiae semen (*Cassia obtusifolia* L. or *Cassia. tora* L.)

The main effects of Cassiae semen are to clear the liver fire, improve eyesight, and moisten the intestine to promote defecation. Modern pharmacological studies have shown that it also has antihypertensive, lipid-lowering, liver-protecting, and anti-oxidative effects (Dong et al., 2021). Liu et al. (2015) established a mouse model of STC and explored the effects of Cassiae semen on colon electromyography activity and the expression of AQP3 in the colonic mucosa of mice with STC. The results showed that Cassia semen can reduce the expression of AQP3 in colonic mucosa and effectively improve colonic motor function in mice. Studies on Cassiae semen have shown that anthraquinones are the main components exhibiting laxative and lipid-lowering effects (Luo et al., 2011; Yu et al., 2022).

#### 4.7.2 Scutellariae Radix (*Scutellaria baicalensis* Georgi)

Baicalin is a flavonoid glycoside and the main active component of Scutellariae Radix. Zheng et al. (2022). recently reported the mechanism underlying the effects of baicalin in the treatment of cerebral ischemia-reperfusion injury and cerebral edema from the perspective of inhibiting astrocyte swelling. It was shown that the expression of glial fibrillary acidic protein (GFAP), transient receptor potential vanilloid 4 (TRPV4), and AQP4 in the cerebral cortex of rats was significantly reduced after treatment with low-dose baicalin. It is hypothesized that baicalin can effectively improve the cerebral edema caused by cerebral ischemia-reperfusion injury in rats. Thus, the effect may be related to the inhibition of astrocyte swelling and the opening of TRPV4 and AQP4 channels. Tu et al. showed that baicalin can inhibit the expression of TNF-α and AQP4 in the brain tissue of rats with ischemic brain injury, reduce the inflammatory response, repair the damaged BBB, and exert a protective effect on ischemic brain injury in rats (Tu et al., 2013).

#### 4.7.3 Picrorhizae Rhizoma (*Picrorhiza scrophulariiflora* Pennell)

Picrorhizae Rhizoma protects the liver, gallbladder, and neurons, and exerts anti-inflammatory and antiviral effects. Picroside II is its main component, exhibiting the highest levels. Studies established a rat model of forebrain ischemia by bilateral common carotid artery ligation to explore the effect of picroside II on the expression of AQP4 in the brain tissue of rats with ischemic injury (Jing et al., 2013; Chen et al., 2016). The results showed that the expression of AQP4 was significantly reduced in the brain tissue of the treatment group. It is hypothesized that picroside II can reduce the damage caused by cerebral ischemia to the BBB and nerve cells by downregulating the expression of AQP4.

### 4.8 Phlegm-dissolving and cough- and panting-relieving medicinals

With dispelling or dispersing phlegm and relieving or alleviating cough and panting as major efficacies, the drugs which are often used to treat phlegm, cough and panting syndromes are called phlegm-dissolving and cough- and panting-relieving medicinals (Zhang and Ye, 2020).

#### 4.8.1 Pinelliae Rhizoma (*Pinellia ternata* (Thunb.) Breit.)

**FIGURE 2:**
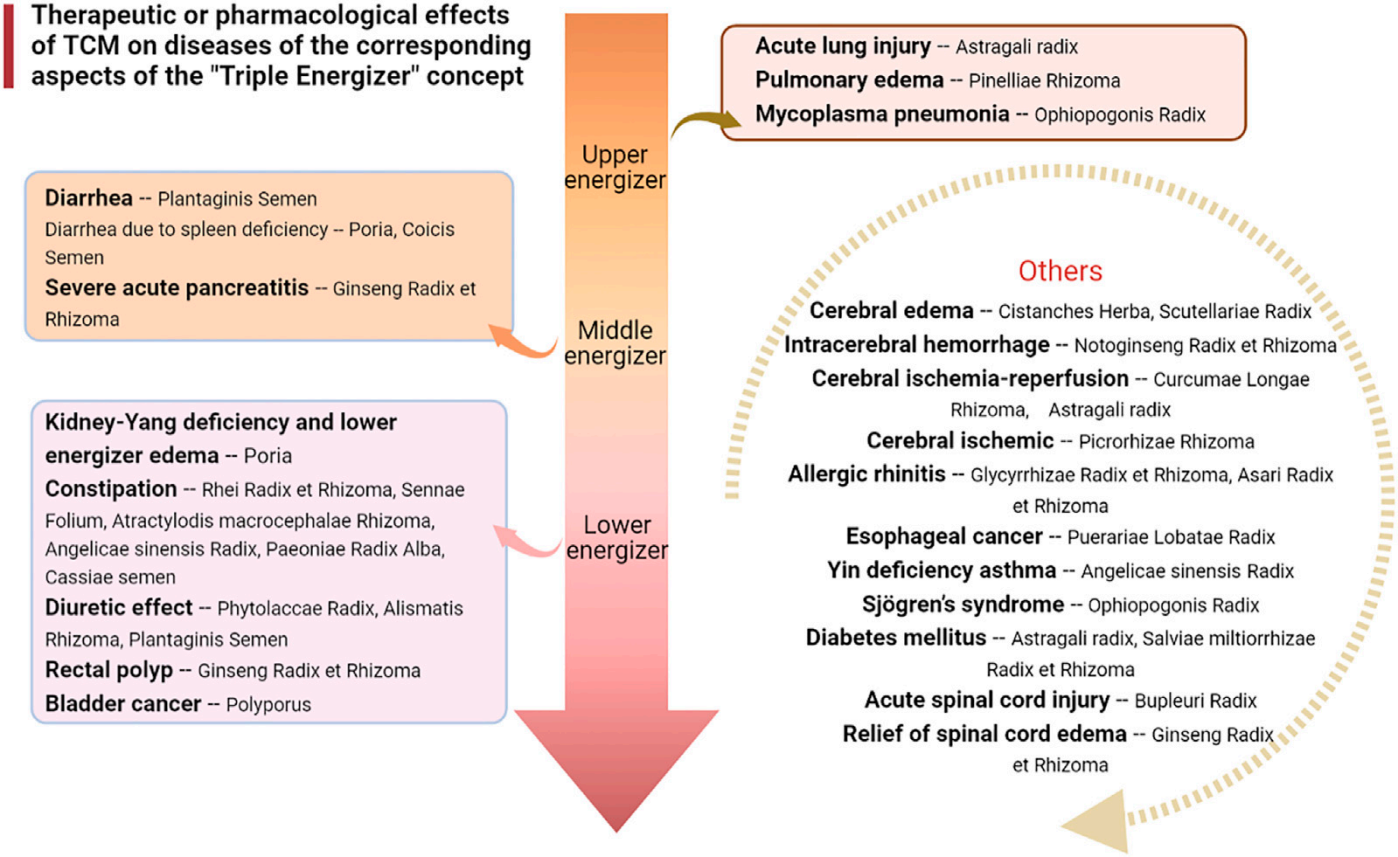
Therapeutic or pharmacological effects of TCM on diseases of the corresponding aspects of the “Triple Energizer” concept.

**FIGURE 3:**
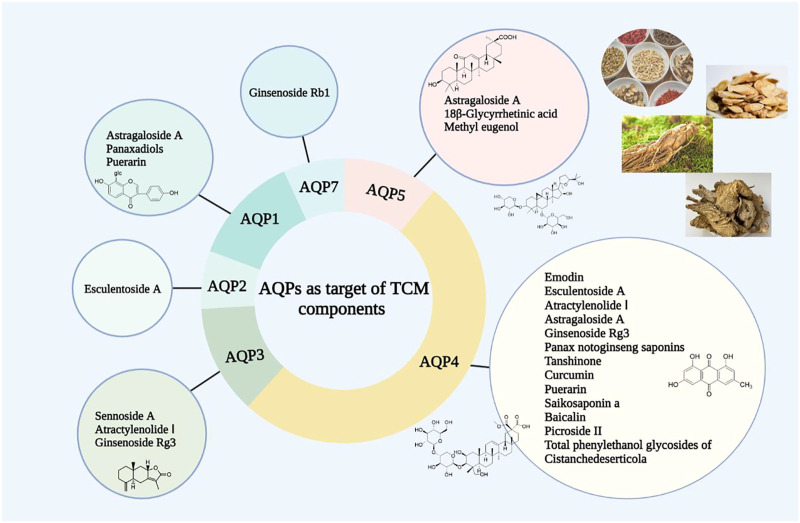
AQPs as target of TCM components.

It has been found that Pinellia extract (EP) or compound Pinellia decoction regulates the expression of AQP in lung tissue (Deng et al., 2009). Zhou et al. explored the pharmacological mechanism of EP in the treatment of ALI (Zhou et al., 2015). The results showed that EP could reduce pulmonary edema by upregulating the expression of AQP5. This mechanism of action may be activated to inhibit the inflammatory factor TNF-α. A new study has also shown that the polysaccharide in aqueous decoction of Pinelliae Rhizoma can reduce the expression of AQP5 mRNA in colon tissue of rats in an asthma model, thus interfering with the asthma process (Huang et al., 2020a) (Figures 2, 3).

## 5 Conclusion

This review article summarizes the active components of TCMs that can regulate AQPs. They are mainly used to treat intestinal disease, diabetes, various types of edema, cerebral ischemia, esophageal cancer, bladder cancer, and other diseases. The therapeutic effects are achieved by upregulating or downregulating the expression of AQPs in related tissues and organs; moreover, these drugs tend to regulate the expression of AQP1-5, especially that of AQP4 (Table 3 and 4). The treatment of TCMs mainly concentrated on the middle energizer and lower energizer. Most AQPs are expressed in the gastrointestinal tract and are involved in the progresses of gastric cancer, colon cancer, diarrhea, constipation, inflammatory bowel disease, and other diseases. Therefore, it is of practical significance to review the current research on the regulation of AQPs to treat digestive system diseases by TCMs and their components.

**TABLE 3:** Regulatory effects of TCM on AQPs.

TCM name	Main classification of triple energizer	Regulatory effects on AQPs	Treatment of disease types/pharmacological effect	Disease model	References
Rhei Radix et Rhizoma	Upper energizer	Downregulation of AQP3 and AQP4 expression in the colon	Constipation	Diarrhea	Bao et al. (2021)
	Middle energizer Lower energizer				
Sennae Folium	Lower energizer	Upregulation of AQP expression in the liver and kidneys, downregulation of AQP expression in the colon	Constipation	Diarrhea	De Ieso and Yool, (2018)
Phytolaccae Radix	Middle energizer Lower energizer	Downregulation of AQP2, AQP4, and mRNA expression in the kidneys	Diuretic effect	Water load	Li et al. (2015)
Atractylodis macrocephalae Rhizoma	Middle energizer Lower energizer	Downregulation of AQP4 expression and upregulation of AQP3 expression in the colon of STC rats	Constipation	STC	Chen et al. (2019)
Astragali radix	Upper energizer	Downregulation of the overexpression of AQP2 in the renal medulla	DM	DM	(Zhao, (2009); Mao et al. (2010); Li et al. (2016))
	Middle energizer	Upregulation of AQP1 and AQP5 expression in the lungs of ALI rats	ALI	ALI	
Ginseng Radix et Rhizoma	Upper energizer	Upregulation of AQP3 and AQP4 expression in rectal polyp tissue	Rectal polyp	Rectal polyp	(Zhao et al. (2009); Li et al. (2019b); Xie et al. (2019b); Xie et al. (2019a); Guo et al. (2020))
	Middle energizer Lower energizer	Upregulation of AQP1 expression in the lungs	Severe acute pancreatitis	——	
		Upregulation of AQP7 and AQP4 expression in the lungs	Improvement of glucose and lipid metabolism		
			Relief of spinal cord edema		
Glycyrrhizae Radix et Rhizoma	Upper energizer	Upregulation of AQP5 expression in nasal mucosa	Allergic rhinitis	Allergic rhinitis	Liu et al. (2020)
	Middle energizer Lower energizer				
Angelicae sinensis Radix	Upper energizer	Downregulation of AQP4 and AQP8 expression in the colon	Constipation	Blood deficiency and constipation	(Wang et al. (2016); Du et al. (2017); Du et al. (2018a); Du et al. (2018b))
	Middle energizer Lower energizer	Upregulation of AQP1 expression in the lungs	Yin deficiency asthma	Asthma	
					
Paeoniae Radix Alba	Middle energizer	Downregulation of AQP4 expression in the colon of STC mice	Constipation	STC	Fang and Gao, (2017)
Cistanches Herba	Lower energizer	Downregulation of AQP4 mRNA expression in the brain tissue of rats with high-altitude cerebral edema	Cerebral edema	High-altitude cerebral edema	(Zhang et al. (2012); Tao et al. (2015))
Ophiopogonis Radix	Upper energizer	Upregulation of AQP5 expression in mice with submandibular gland inflammation	SS	SS	(Tao et al. (2014); Sun et al. (2021b))
	Middle energizer	Upregulation of AQP1 and AQP5 expression in the lung tissue of mice with mycoplasma pneumonia	Mycoplasma pneumonia		
Poria	Upper energizer	Downregulation of AQP1 and AQP2 expression in the renal medulla of rats with kidney-Yang deficiency and lower energizer edema	Kidney-Yang deficiency and lower energizer edema	Kidney-Yang deficiency and lower energizer edema	(Zhang, (2012); Li et al. (2021a))
	Middle energizer Lower energizer	Upregulation of AQP3 expression in colonic mucosa	Diarrhea due to spleen deficiency	Diarrhea due to spleen deficiency	
Alismatis Rhizoma	Lower energizer	Downregulation of AQP2 mRNA expression in the renal medulla	Diuretic effect	——	Wu et al. (2010)
Polyporus	Lower energizer	Downregulation of AQP1 and AQP3 mRNA expression in rats with bladder cancer	Bladder cancer	Combination of BBN with saccharin to induce Fisher-344 rat bladder cancer	(Zhang et al. (2011); Zhang and Zhang, (2013))
Coicis Semen	Upper energizer	Upregulation of AQP3 expression in colon tissue	Dampness stagnancy due to spleen deficiency	Dampness stagnancy due to spleen deficiency	Wang et al. (2018b)
	Middle energizer				
Plantaginis Semen	Upper energizer	Downregulation of AQP1 and AQP2 expression in the renal medulla	Diuretic effect	Water load	(Yan et al. (2014); Wang et al. (2020c); Wang et al. (2020b))
	Middle energizer Lower energizer	Upregulation of AQP1 and AQP4 expression in intestinal tissue	Diarrhea	Diarrhea	
Notoginseng Radix et Rhizoma	Middle energizer	Downregulation of AQP4 and AQP4 mRNA expression	Intracerebral hemorrhage	Intracerebral hemorrhage	Meng et al. (2007)
Salviae miltiorrhizae Radix et Rhizoma	Upper energizer	Downregulation of AQP2 expression in kidney tissue of diabetic rats	DM	DM	Hu et al. (2011)
		Upregulation of AQP1 expression in the lungs			
Curcumae Longae Rhizoma	Middle energizer	Downregulation of AQP4 expression	Focal cerebral ischemia-reperfusion	Focal cerebral ischemia-reperfusion	Lin, (2015)
Puerariae Lobatae Radix	Upper energizer	Inhibition of AQP4 expression	Subarachnoid hydrops	Subarachnoid hemorrhage	(Jiang et al. (2011); Wang et al. (2018a); Ying and Liu, (2020))
	Middle energizer	Upregulation of AQP1 mRNA expression	Esophageal cancer		
Bupleuri Radix	Upper energizer	Downregulation of AQP4 expression	Acute spinal cord injury	Acute spinal cord injury	Zhu et al. (2017a)
	Middle energizer				
Asari Radix et Rhizoma	Upper energizer	Upregulation of AQP5 expression in the nasal mucosa	Allergic rhinitis	Allergic rhinitis	Wu et al. (2019)
	Lower energizer				
Cassiae semen	Lower energizer	Downregulation of AQP3 expression colonic mucosa	Constipation	STC	Liu et al. (2015)
Scutellariae Radix	Upper energizer	Downregulation of AQP4 expression in brain tissue	Cerebral ischemia-reperfusion injury Cerebral edema	Cerebral ischemic injury	(Abir-Awan et al. (2019); Zheng et al. (2022))
	Middle energizer				
Picrorhizae Rhizoma	Middle energizer Lower energizer	Downregulation of AQP4 expression	Cerebral ischemic	Forebrain ischemia by bilateral common carotid artery ligation	Jing et al. (2013)
Pinelliae Rhizoma	Upper energizer	Upregulation of AQP5 expression	Pulmonary edema	ALI	(Zhou et al. (2015); Huang et al. (2020a))
	Middle energizer	Downregulation of AQP5 mRNA expression in colon tissue of rats	Asthma	Asthma	

Abbreviations: TCM, traditional chinese medicine; AQP, aquaporin; STC, slow transit constipation; DM, diabetes mellitus; ALI, acute lung injury; SS, Sjögren’s syndrome; BBN, N-butyl-N-(4-hydroxybutyl)-nitrosamine.

**TABLE 4:** Chemicals used in TCM and targeted AQP.

Chemical	TCM name	Chemical structure	Targeted AQP	References
**Emodin**	Rhei Radix et RhizomaCassiae semen	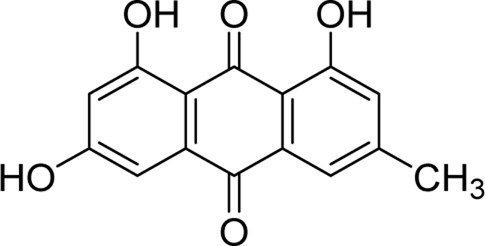	AQP4	Peng et al. (2017)
**Sennoside A**	Rhei Radix et RhizomaSennae Folium	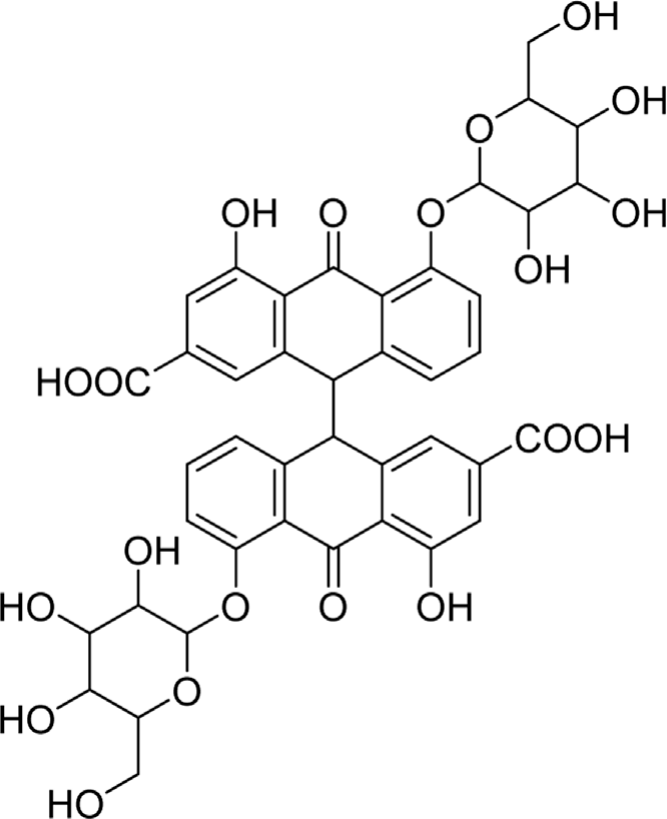	AQP3	Kon et al. (2014)
**Esculentoside A**	Phytolaccae Radix	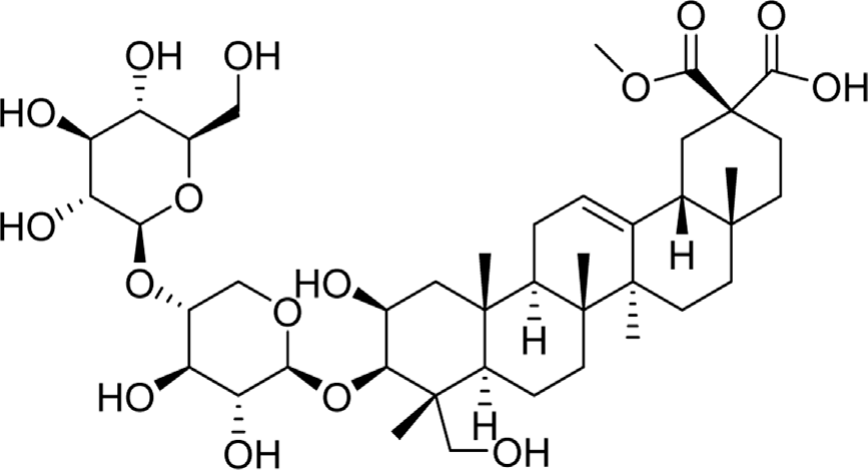	AQP2AQP4	Li et al. (2015)
**Atractylenolide Ⅰ**	Atractylodis macrocephalae Rhizoma	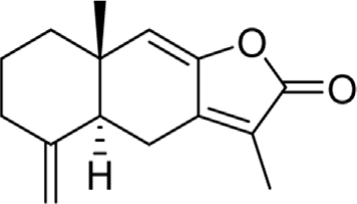	AQP3AQP4	Jiang, (2017)
**Astragaloside A**	Astragali radix	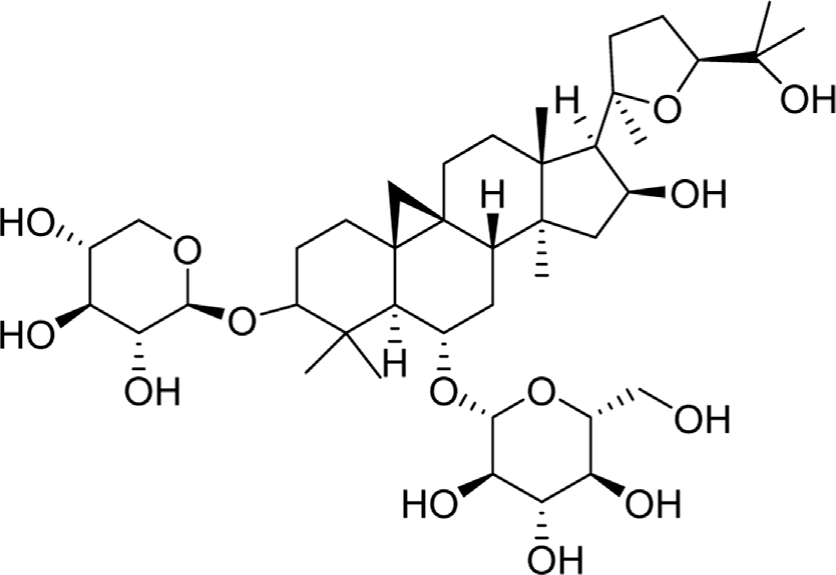	AQP1AQP4AQP5	(Zhao, (2009); Li et al. (2016))
**Ginsenoside Rg3**	Ginseng Radix et Rhizoma	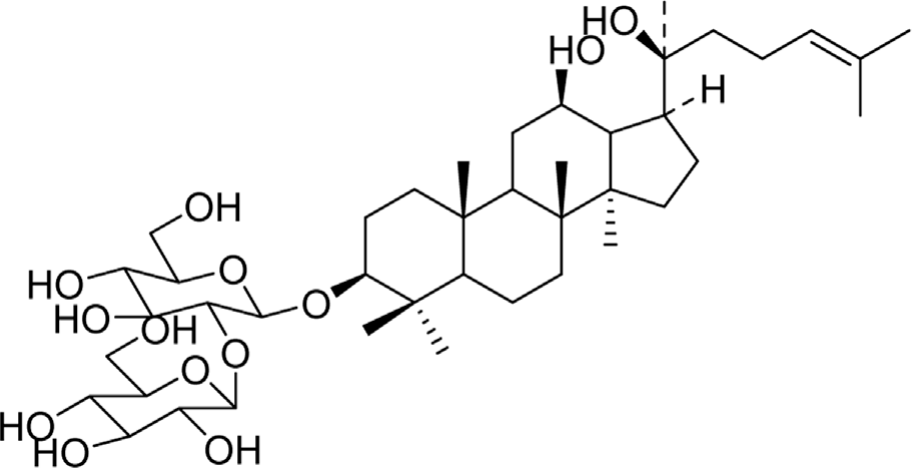	AQP3AQP4	(Xie et al. (2019b); Xie et al. (2019a))
**Panaxadiols**	Ginseng Radix et Rhizoma	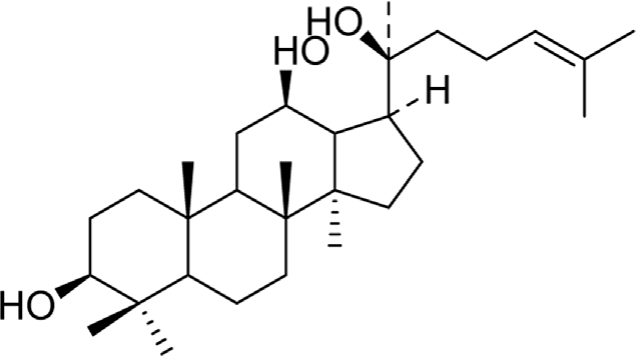	AQP1	Zhao et al. (2009)
**Ginsenoside Rb1**	Ginseng Radix et Rhizoma	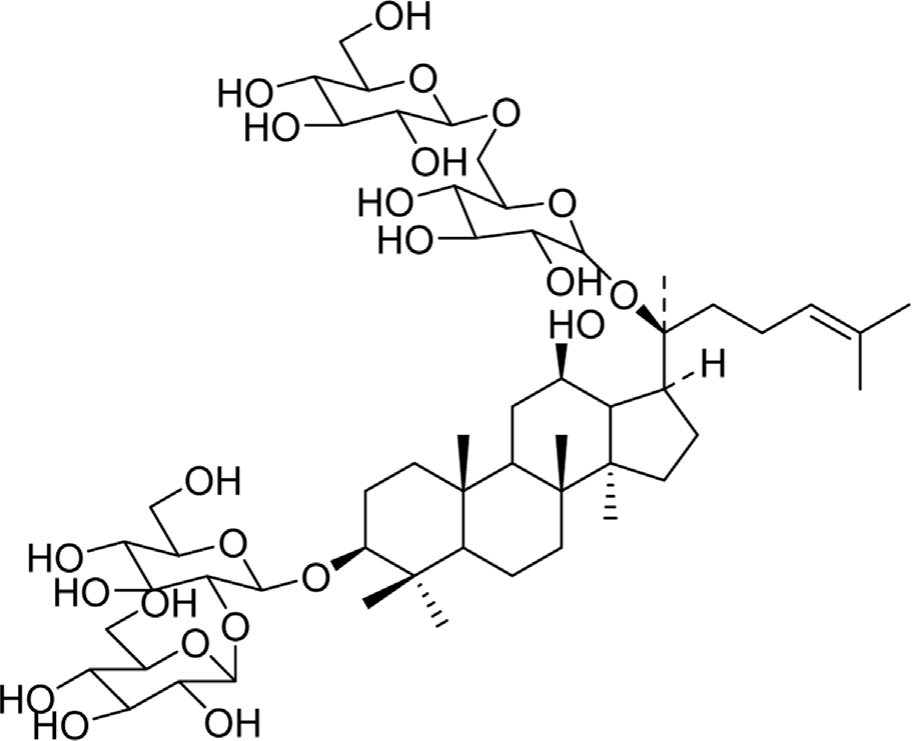	AQP7	Guo et al. (2020)
**18β-glycyrrhetinic acid**	Glycyrrhizae Radix et Rhizoma	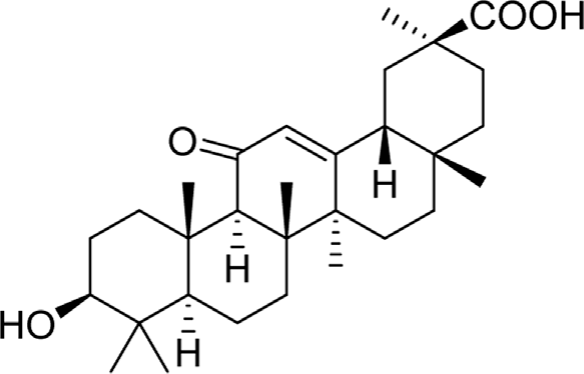	AQP5	Liu et al. (2020)
**Tanshinone**	Salviae miltiorrhizae Radix et Rhizoma	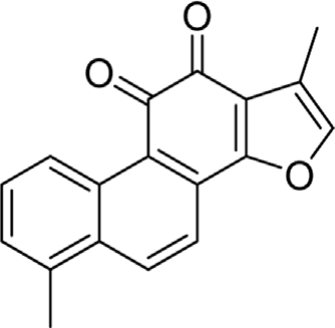	AQP4	Tang et al. (2022)
**Curcumin**	Curcumae Longae Rhizoma	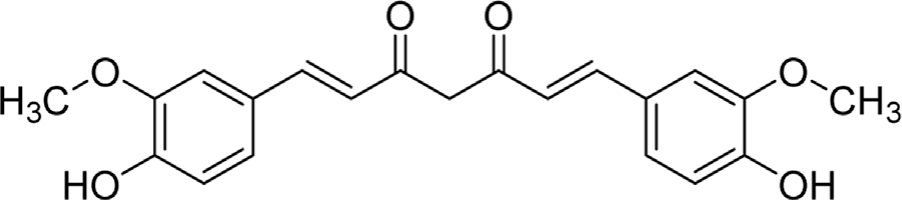	AQP4	Lin, (2015)
**Puerarin**	Puerariae Lobatae Radix	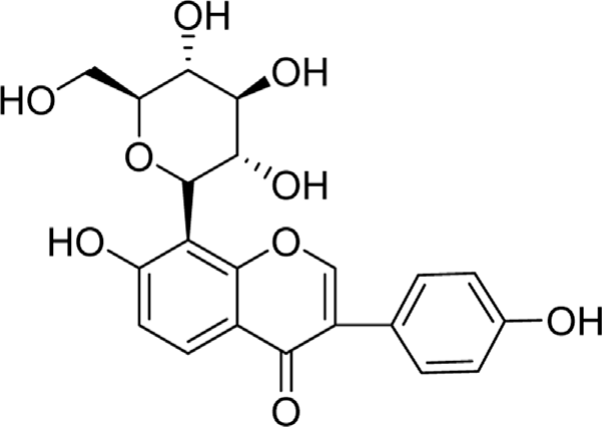	AQP1AQP4	(Jiang et al. (2011); Wang et al. (2018a))
**Saikosaponin a**	Bupleuri Radix	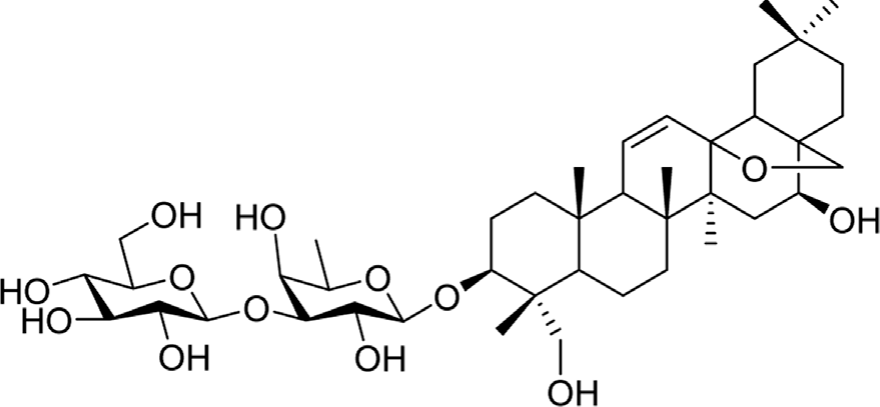	AQP4	Zhu et al. (2017a)
**Methyl eugenol**	Asari Radix et Rhizoma	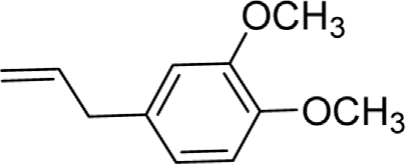	AQP5	Wu et al. (2019)
**Baicalin**	Scutellariae Radix	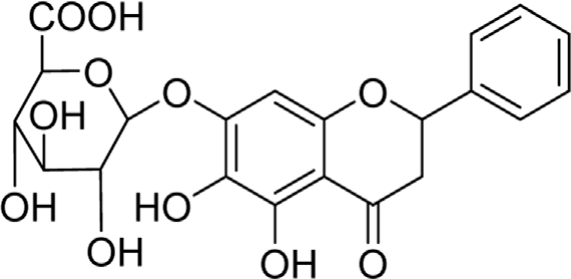	AQP4	(Tu et al. (2013); Zheng et al. (2022))
**Picroside II**	Picrorhizae Rhizoma	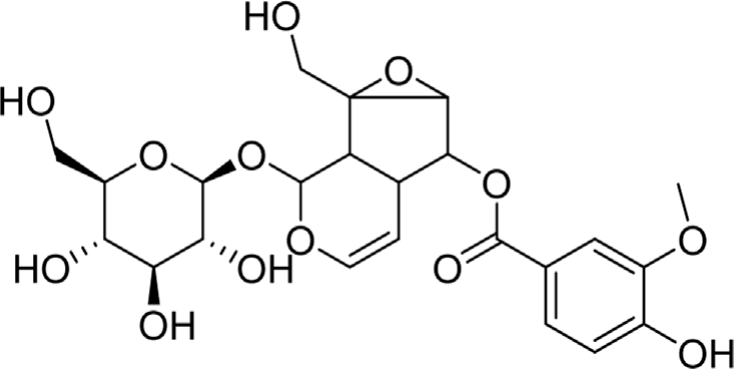	AQP4	Jing et al. (2013)

First, abundant studies have proved that the expression of AQPs is related to digestive system tumors, such as gastric cancer, colorectal cancer, esophageal cancer, liver cancer, and pancreatic cancer. There is also evidence that TCM can treat gastrointestinal tumor disease by regulating AQP expression, such as Puerariae Lobatae Radix and its components can treat esophageal cancer. However, the treatment of AQP-related diseases by TCMs and their components mentioned in our review have only been assessed in preliminary pharmacological studies, and there are not yet adequate data on the mechanisms of TCM regulating AQPs to treat digestive system diseases. Second, there is limited evidence regarding the pharmacokinetics, toxicity, and clinical efficacy of these reported TCMs or their components. At present, research on the regulation of AQPs is focused more on the combination of TCMs, rather than on treatment with a single TCM. Therefore, despite continuous progress in this field, additional studies are needed to develop new drugs from these medicines for the treatment or prevention of diseases. In summary, the specific mechanisms involved in the regulation of AQPs by TCMs and their components warrant further investigation. This may make TCMs or AQP regulators become a new treatment for digestive diseases. We hope this review may provide directions for the development of new drugs from TCMs for the treatment of diseases caused by abnormal water metabolism.

## Abbreviations

ALI, Acute lung injury; AQP, Aquaporin; BBB, Blood-brain barrier; BBN, N-butyl-N-(4-hydroxybutyl)-nitrosamine; DM, Diabetes mellitus; EP, Pinellia extract; PNS, Panax notoginseng saponins; RT-PCR, Reverse transcription polymerase chain reaction; SS, Sjögren’s syndrome; SSa, Saikosaponin a; STC, Slow transit constipation; TCM, Traditional Chinese medicine; CM, Chinese medicine; TRPV4, Transient receptor potential vanilloid four; VEGF, Vascular endothelial growth factor.
